# Young Investigator Award 2026: Development of a reconstructed induced pluripotent stem cell-derived immunocompetent skin model

**DOI:** 10.1007/s00210-026-05652-8

**Published:** 2026-07-06

**Authors:** Marla Dubau

**Affiliations:** 1 Department of Pharmacology and Toxicology, Institute of Pharmacy, Freie Universität Berlin, Königin-Luise Str. 2+4, 14195 Berlin, Germany; 2 Present Address: German Federal Institute for Risk Assessment, Department of Pesticides Safety, Berlin, Germany

**Keywords:** Induced pluripotent stem cells, Skin sensitization, Immunocompetent skin model, Reconstructed human skin, Adverse outcome pathway

## Abstract

Skin sensitization testing is moving towards human-relevant, non-animal approaches, yet many current in vitro methods address only individual key events of the adverse outcome pathway. Here, a reconstructed immunocompetent human skin model is presented, generated entirely from induced pluripotent stem cell-derived fibroblasts, keratinocytes, and dendritic cells. The model reproduced key structural features of human skin and enabled the combined assessment of keratinocyte activation and dendritic-cell maturation and migration following topical exposure to skin sensitizers. By integrating epidermal and immune responses within a single three-dimensional skin construct, this model provides a mechanistically informative platform for skin sensitization research and supports the development of ethically responsible testing strategies.

Skin sensitization is an important toxicological endpoint in the safety assessment of pharmaceuticals, chemicals, and cosmetics (Tourneix et al. 2019). Historically, hazard identification has relied on animal-based assays, such as the guinea pig maximization test and Buehler assay (OECD 2022) and the local lymph node assay–based methods (OECD 2010). However, these methods are increasingly challenged by ethical concerns, regulatory limitations, and incomplete predictive value for human biology (Basketter et al. 2009; Natsch et al. 2023). In parallel, the adverse outcome pathway (AOP) framework and OECD-guidance for non-animal skin sensitization testing have advanced the development of non-animal testing strategies (Kolle et al. 2020; OECD 2012). Nevertheless, many currently available in vitro methods still focus on individual key events of the AOP and therefore do not fully reproduce the interaction between epidermal and immune cells that characterizes the skin’s response to skin sensitizing substances.

The present work addressed this gap by developing a reconstructed human skin model composed entirely of induced pluripotent stem cell (iPSC)-derived cell populations. The model integrated iPSC-derived fibroblasts, keratinocytes, and dendritic cells into a three-dimensional (3D), immunocompetent skin model designed for the assessment of skin-sensitizing substances. In contrast to conventional reconstructed skin equivalents that focus mainly on tissue architecture or barrier properties, this system combined structural organization with an active immune component by incorporating iPSC-derived dendritic cells and thereby allowed the investigation of both keratinocyte-driven and dendritic-cell-driven responses (key events 2 and 3 of the AOP for skin sensitization) within a single assay (Dubau et al. 2025).

Human hair follicle-derived keratinocytes served as the starting material for reprogramming and were converted into iPSC using non-integrative Sendai viral vectors carrying the four Yamanaka factors. The resulting iPSC were subsequently differentiated into fibroblasts, keratinocytes, and dendritic cells using lineage-specific protocols. This approach provided a renewable and standardized source of human cell types, reduced reliance on repeated primary tissue isolation, and mitigated important limitations associated with primary cells and immortalized cell lines. The differentiated cells exhibited properties consistent with their intended lineages. iPSC-derived fibroblasts supported extracellular matrix formation, iPSC-derived keratinocytes showed features of epidermal lineage commitment and barrier-associated differentiation, and iPSC-derived dendritic cells displayed a mature antigen-presenting phenotype and retained immune functionality.

To generate the immunocompetent skin model, iPSC-derived fibroblasts were embedded in a collagen matrix on a porous membrane support. After dermal solidification, iPSC-derived keratinocytes and dendritic cells were added to the surface to establish the epidermal compartment. This approach yielded a full-thickness reconstructed tissue with integrated immune cells, in contrast to many published co-culture skin models in which monocyte-derived dendritic cells or monocyte-derived Langerhans cells are only partially incorporated into the 3D structure (Chau et al. 2013; Schellenberger et al. 2019). Histological and immunofluorescence analyses demonstrated that the developed tissue reproduced key structural features of human skin. Tight junction formation was indicated by claudin staining, which was particularly prominent in the epidermal layer. Filaggrin was detected in the outer epidermal region, indicating barrier-associated differentiation. Additional epidermal markers included cytokeratin 10, cytokeratin 14, and involucrin, confirming the presence of differentiated suprabasal and basal keratinocyte populations. Within the epidermal compartment, integrated iPSC-derived dendritic cells were identified by expression of HLA-DR, CD86, and Dectin-1. Together, these findings showed that the skin model combined tissue architecture, epidermal differentiation, and immune-cell incorporation in a single 3D construct.

A central aim of the work was to determine whether the skin model could respond to sensitizing chemicals. For this purpose, the reconstructed tissues were exposed topically for 24 h to compounds of different sensitization potency, namely 2,4-dinitrochlorobenzene, p-phenylenediamine, isoeugenol, and resorcinol, while glycerol served as a non-sensitizing control. Following treatment, iPSC-derived dendritic cells migrated out of the skin model into the lower compartment in significantly increased numbers after stimulation with a sensitizing substance. Flow-cytometry analysis of the migrated cells demonstrated increased expression of the dendritic cell characterization and antigen-presentation-associated markers CD209, HLA-DR, and CD86 compared with untreated and glycerol-treated controls. These results indicate that the integrated dendritic cells remained functional within the tissue and responded to sensitizing agents by undergoing maturation and migration, which are hallmark features of key event 3 of the skin sensitization adverse outcome pathway.

To assess inflammatory signaling associated with skin sensitization, cytokine release from the immunocompetent skin model was analyzed after topical exposure to the test substances. Cytokine profiling (IL-8, IL-1β, MIP-1β, TGF-β, IL-18, MMP-9, TSLP) showed robust increases in IL-8, IL-1β, MIP-1β, and TGF-β after exposure to 2,4-dinitrochlorobenzene and p-phenylenediamine, while isoeugenol and resorcinol elicited more moderate and selective responses. IL-18 increased significantly after isoeugenol and resorcinol exposure, whereas increases in the keratinocyte-associated mediators MMP-9 and TSLP were most pronounced after treatment with 2,4-dinitrochlorobenzene and p-phenylenediamine, respectively. Glycerol did not induce comparable changes.

An important strength of this skin model lies in its ability to capture more than one key event of the skin sensitization pathway of the AOP within a single assay. The cytokine and mediator profile reflects keratinocyte activation, corresponding to key event 2, while dendritic-cell migration and maturation represent key event 3. This integrated design distinguishes the platform from assay batteries in which separate test systems must be combined to address multiple key events of the AOP for skin sensitization.

Taken together, the results establish a reconstructed immunocompetent skin model generated entirely from iPSC-derived cells. The model reproduced essential structural features of human skin, maintained viability under test conditions, supported integration of functional dendritic cells, and responded to sensitizers with biologically relevant maturation and cytokine secretion. By enabling the simultaneous assessment of keratinocyte activation and dendritic-cell activation in one 3D human tissue, this work advances current non-animal approaches for skin sensitization testing and provides a mechanistically informative platform for toxicological research. The study therefore contributes to the development of more human-relevant and ethically responsible strategies for skin sensitization assessment (Fig. 1).

**Fig. 1:**
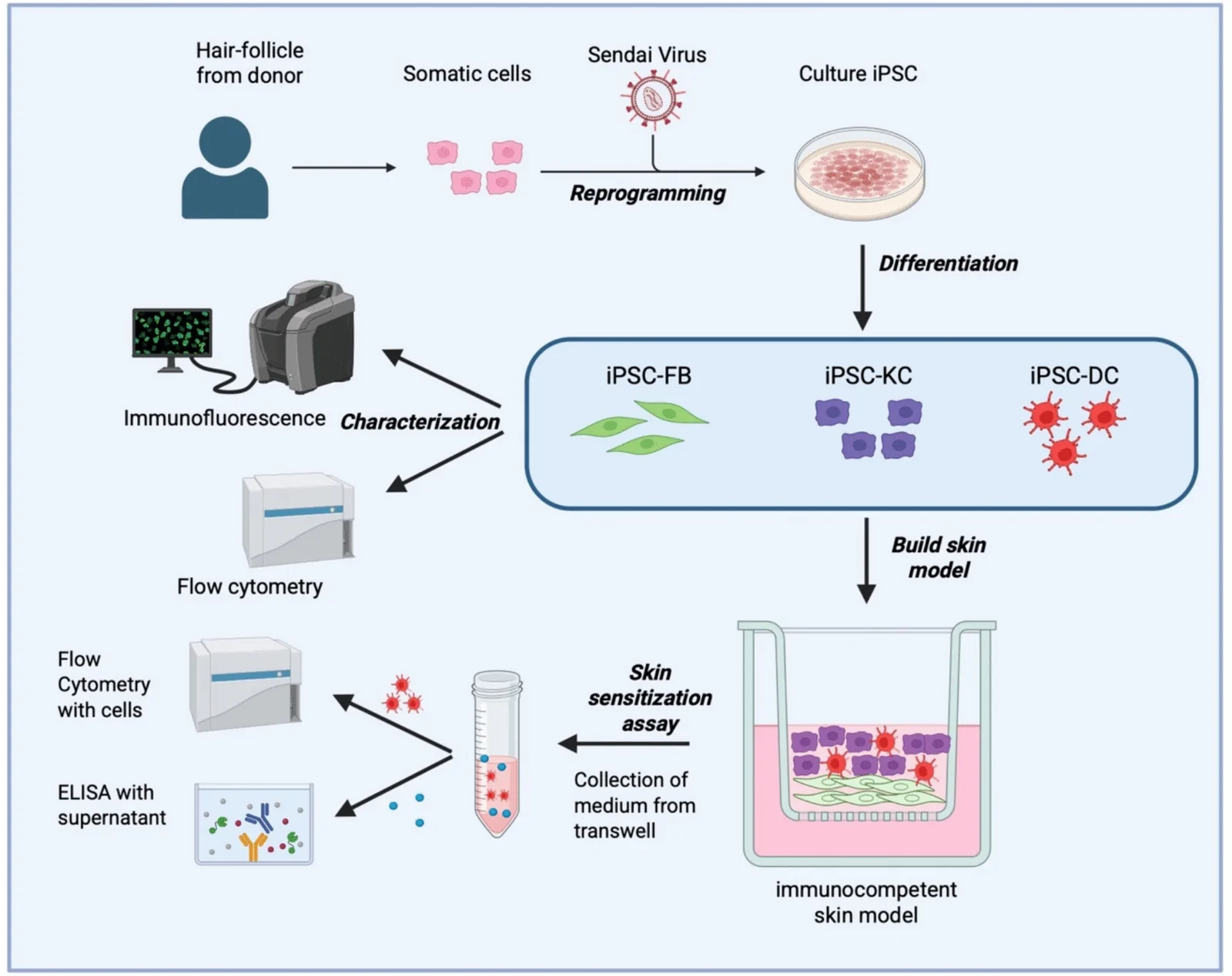
Schematic overview of the generation and application of a fully iPSC-derived immunocompetent reconstructed human skin model (RHS) for skin sensitization assessment. Hair follicle-derived keratinocytes are reprogrammed to iPSC using non-integrative Sendai virus vectors and subsequently differentiated into iPSC-derived fibroblasts (iPSC-FB), keratinocytes (iPSC-KC), and dendritic cells (iPSC-DC). Following cellular characterization (immunofluorescence and flow cytometry), the cell types are assembled into a three-dimensional RHS for topical exposure to test substances. Sensitization-associated readouts include analysis of migrated cells by flow cytometry and cytokine quantification in culture supernatants by ELISA. Illustration created with BioRender.com. Reproduced from: Dubau M. Development of a Reconstructed Induced Pluripotent Stem Cell-Derived Immunocompetent Skin Model. Doctoral thesis, Freie Universität Berlin Refubium, 2025. Licensed under CC BY 4.0

## Data Availability

Data described in the manuscript is available upon request from the corresponding author.
